# Leukocytosis in acute stroke: A prospective study from an emergency department setting

**DOI:** 10.12669/pjms.42.9.14805

**Published:** 2026-09

**Authors:** Muhammad Abas Khan

**Affiliations:** 1 Dr. Muhammad Abas Khan Assistant Professor, Emergency Department, Lady Reading Hospital, Peshawar, Pakistan

**Keywords:** Hemorrhagic Stroke, Ischemic Stroke, Leukocytosis, Inflammation, Stroke, Total Leukocyte Count

## Abstract

**Objectives:**

To determine the total leukocyte count (TLC) in patients with acute stroke and assess its association with stroke type and age.

**Methodology:**

This prospective observational study was conducted at the Department of Emergency Medicine, Lady Reading Hospital, Peshawar, from May to November 2025. Adult patients with acute ischemic or hemorrhagic stroke were enrolled by non-probability consecutive sampling. TLC was measured at presentation, and age and stroke subtype were recorded. Patients with active infections, inflammatory diseases, hematological disorders, or immunosuppressive therapy were excluded. Data were analyzed using SPSS version 23. A one sample *t-test* was applied to compare the mean TLC value of the study population with the international reference mean TLC value of 7×10^9^/L. Chi-square assessed the association between TLC category and stroke type. One-way ANOVA compared TLC across age groups (30–49, 50–69, ≥70 years), and Pearson correlation assessed the relationship between age and TLC. A p-value <0.05 was considered significant.

**Results:**

Ninety patients were included. Mean TLC was significantly higher than the reference value (11.95±4.62×10^9^/L; p<0.001). No significant association was found between TLC category and stroke type (p=0.399). TLC did not differ significantly across age groups (p=0.119), and no significant correlation was observed between age and TLC (r=-0.108; p=0.313).

**Conclusion:**

TLC was elevated in acute stroke but showed no significant association with stroke subtype or age. Although leukocytosis is common in acute stroke, TLC alone has limited value in differentiating stroke types. Larger studies are needed to evaluate its prognostic significance.

## INTRODUCTION

Stroke remains one of the leading causes of mortality and long-term disability worldwide, imposing a major clinical and economic burden on healthcare systems.^1^ Despite advances in diagnostic imaging and therapeutic strategies, early risk stratification and prognosis in acute stroke continue to present challenges, particularly in low- and middle-income countries.^2^,^3^ Inflammation has emerged as a critical component in the pathophysiology of stroke, influencing neuronal injury, edema formation, and secondary ischemic damage.^4^

Among the various inflammatory biomarkers, the total leukocyte count (TLC) is routinely available, inexpensive, and widely used in emergency settings. Elevated TLC at presentation has been associated with worse neurological outcomes, larger infarct sizes, and increased mortality in several studies.^5^,^6^ Furthermore, and leukocytosis may reflect the underlying systemic stress response triggered by acute cerebrovascular events, irrespective of stroke subtype.^7^ However, the magnitude of leukocyte elevation can vary across populations due to demographic, environmental, and genetic factors, highlighting the need for region-specific data.^8^

In Pakistan, limited research has explored the behavior of TLC in acute stroke patients, particularly its distribution and comparison with international reference values. Additionally, evidence regarding the relationship between TLC and factors such as age or stroke subtype remains inconsistent in the local context.^9^

Globally, stroke represents a catastrophic public health crisis, causing approximately 7.3 million deaths annually and ranking as the second leading cause of mortality worldwide.^2^ In 2021, the Global Burden of Disease study recorded 11.9 million new incident stroke cases and over 93.8 million stroke survivors registered globally.^2^ The global absolute number of incident strokes has risen substantially by 70.0% between 1990 and 2021. Stroke-related deaths have also experienced a significant surge of 44.0% over this same thirty-year period.^2^ Pathologically, ischemic stroke constitutes approximately 65.3% of these incident cases, while intracerebral hemorrhage accounts for 28.8% of cases^2^. Low- and middle-income countries bear a disproportionately heavy portion of this burden, accounting for 87.0% of all stroke-related deaths.^2^ These developing nations are also responsible for 89.0% of the total disability-adjusted life years lost to stroke worldwide.^2^ Approximately 84.0% of this heavy global stroke burden is directly attributable to 23 modifiable metabolic, behavioral, and environmental risk factors.^2^ The estimated global cost of stroke care now exceeds US$890 billion per year, which translates to roughly 0.66% of the global gross domestic product.^2^

Within the vast territories of Asia, the stroke burden is exceptionally high as the continent houses over 60.0% of the global population.^3^ The age-standardized stroke incidence shows immense regional variation across Asian nations, ranging from a high of 226.4 per 100,000 people to a low of 96.0 per 100,000.^4^ South Asia alone accounts for over 40.0% of global stroke-related fatalities, representing a severe public health crisis for its 1.9 billion inhabitants.^5^ Clinical registries indicate that large artery atherosclerosis and small artery occlusion represent the primary stroke mechanisms in over 75.0% of Asian patients.^5^ In rural communities of Bangladesh, recent epidemiological investigations have reported a baseline stroke prevalence rate of 1.07 per 1,000 people.^6^

At the national level, stroke represents the second leading cause of mortality in Pakistan, which has a population of 225 million.^6^ In 2021, stroke-related events were responsible for 99,759 deaths across the provinces of Pakistan.^7^ The GBD 2021 database estimates that Pakistan has an age-standardized stroke incidence rate of 153 and a prevalence rate of 1,088 per 100,000 population.^7^ Subnationally, a systematic integrated population health survey in Khyber Pakhtunkhwa revealed a stroke prevalence of 1.2% in adults.^8^ In Punjab, Pakistan’s most populous province, a comprehensive hospital-based analysis showed that stroke represents 1.03% of all 159,824 emergency department visits.^6^ Within this emergency cohort, male patients constitute 51.75% and female patients constitute 48.25%, with an overall average age of 57.19 years.^4^ Co-morbidities are highly prevalent in this emergency department setting, with hypertension observed in 61.63% and diabetes in 17.65% of the stroke population.^6^ Crucially, a significant 62.39% of these emergency admissions are discharged with a major functional disability.^6^ In similar South Asian emergency facilities, the overall in-hospital mortality rate for stroke patients is reported at 11.7%, with deaths occurring at a mean of 5.9 days post-admission.^9^

Pathophysiologically, acute ischemic events trigger a rapid systemic immune-inflammatory cascade, leading to leukocytosis in up to 38.5% of patients at admission.^10^ This reactive leukocytosis is characterized by an absolute white blood cell count exceeding 11,000 cells per microliter in adults^11^. Observational research involving 14,174 patients has shown that elevated white blood cell counts are associated with a 1.7-fold increase in all-cause mortality.^10^ This baseline leukocyte elevation is also independently linked to a 1.55-fold higher risk of long-term mortality and a 1.61-fold increased risk of recurrent vascular events.^10^ The underlying cellular mechanism involves the rapid mobilization of bone marrow neutrophils, which represent 50.0% to 70.0% of circulating white blood cells.^11^ Within 24 to 72 hours of stroke onset, these activated neutrophils infiltrate the ischemic brain parenchyma, where they secrete matrix metalloproteinase-9, causing blood-brain barrier disruption.^6^,^10^ This neutrophil recruitment is accompanied by a decline in lymphocytes, yielding an elevated neutrophil-to-lymphocyte ratio that increases the risk of poor 90-day functional outcomes by 1.15-fold per unit increase.^9^ A high neutrophil-to-lymphocyte ratio at day one is also a strong predictor of symptomatic intracranial hemorrhage, with a receiver operating characteristic cutoff value of 5.1 demonstrating a sensitivity of 57.9%.^6^ Furthermore, this systemic inflammatory stress, quantified by the Endothelial Activation and Stress Index in a cohort of 786 severe stroke patients, is associated with a 1.11-fold increase in 28-day all-cause mortality.^11^

Although several studies have reported an association between admission leukocytosis and adverse outcomes in acute stroke, the findings remain inconsistent. Some investigators identified leukocytosis as an independent predictor of mortality and poor functional recovery, whereas others observed that the association weakened after adjustment for stroke severity, concurrent infection, or comorbid conditions. In addition, relatively few studies have directly compared leukocyte responses between ischemic and hemorrhagic stroke or explored whether these responses vary across different age groups.

Evidence from South Asia, particularly Pakistan, is limited. Most available studies are retrospective, originate from developed countries, or focus exclusively on ischemic stroke. Prospective data from emergency department settings evaluating leukocytosis across stroke subtypes and different age groups remain scarce. Therefore, the present study was designed to determine the frequency of leukocytosis among patients presenting with acute stroke, compare leukocyte counts according to stroke subtype and age, and contribute locally relevant evidence that may improve early risk stratification and clinical decision-making.

## METHODOLOGY

This prospective observational study was conducted in the Department of Emergency Medicine, lady reading Hospital Peshawar from May to November 2025. A total of 90 patients were included in this study who presented with acute stroke in emergency department of lady reading hospital Peshawar. All the patients were females.

### Ethical approval:

It was obtained from the Institutional Review Board on April 15, 2025 (Approval No: 278/LRH/MTI).

The sample size was calculated with the help of who sample size calculator 1.1 with the confidence interval of 95%. The sample size was calculated from 37.2% and anticipated population proportion of patients with increased total leucocyte count in stroke patients of 0.372.absolute precision required was 10%. Frequency of total leucocyte count in stroke patients for the sample size was taken from a reference study conducted by Vo TP et al.^10^ A non-probability consecutive sampling technique was used. All eligible stroke patients presenting during the study period were included until the required sample size was achieved.

### Inclusion criteria:

- Patients aged ≥18 years and Diagnosed with acute stroke (ischemic or hemorrhagic) based on clinical assessment and neuroimaging were included in the study. Also the Patients included in the study were those who presenting within the first 24 hours of symptom onset and Patients who consented to participate in the study.^12^

### Exclusion Criteria:

- This study excludes male patients presenting with stroke symptoms, alongside anyone experiencing a stroke mimic or a trauma-related brain injury. To ensure accurate data, patients with active infections (such as sepsis, pneumonia, or urinary tract infections), chronic inflammatory disorders, or hematological diseases that alter leukocyte levels are also ineligible. Finally, individuals currently prescribed steroids, immunosuppressive therapies, or cytotoxic drugs are excluded from the cohort. The exclusion criteria were determined based on a detailed medical history, clinical examination, review of available medical records, current medication history, and routine laboratory investigations performed at the time of presentation.
- A structured proforma was utilized to systematically document all relevant patient data during the study proceedings. This included comprehensive demographic information (age and gender), stroke classification (ischemic versus hemorrhagic), and initial laboratory findings, specifically the total leukocyte count (TLC) obtained at the time of presentation. For sub-analysis, age was stratified into three distinct cohorts (30–49 years, 50–69 years, and ≥70 years).Total leukocyte count was measured from venous blood samples collected at the time of presentation to the Emergency Department before initiation of treatment. An elevated TLC was defined as a value >11 ×10^9^/L according to the standard adult laboratory reference range of 4–11 ×10^9^/L. Additionally, the proforma captured the exact time of symptom onset, specific presenting clinical manifestations of the stroke, and a comprehensive medical history/screening checklist used to strictly exclude active infections or chronic inflammatory disorders. Finally, neuroimaging details from brain CT or MRI scans were recorded to confirm the definitive stroke subtype and verify the absence of traumatic brain injuries or stroke mimics.
- For categorical analysis, TLC values were classified as normal (4–11 ×10^9^/L) or elevated (>11 ×10^9^/L) according to the standard adult reference range reported in the literature.

### Statistical analysis:

Data were analyzed using SPSS version 23. Descriptive statistics (mean, median, mode, standard deviation) were calculated for continuous variables. A one-sample t-test was used to compare mean TLC with the international reference value The chi-square test was applied to assess the association between two categorical variables: TLC category (normal vs elevated) and stroke type (ischemic vs hemorrhagic). A one-way ANOVA was used to compare mean TLC across the three age groups. Pearson correlation analysis was performed to assess the relationship between age and TLC. A p-Value <0.05 was considered statistically significant.

## RESULTS

A total of 90 stroke patients were included in the study. The mean age of the participants was 61.1 ± 15.1 years (range: 25–100 years). The mean total leukocyte count (TLC) was 11.95 ± 4.62 ×10^9^/L (range: 3.87–30.40 ×10^9^/L). The median TLC was 11.2 ×10^9^/L, and the mode was 11 ×10^9^/L (Table-I). A one-sample t-test demonstrated that the mean TLC of stroke patients was significantly higher than the reference mean value of 7 ×10^9^/L (mean difference = 4.95 ×10^9^/L; 95% CI: 3.98–5.92; t = 10.17, df = 89, p < 0.001), indicating that stroke patients had significantly elevated leukocyte counts compared with the reference value (Table II).

**Table-I:** Descriptive statistics of total leucocyte count (×10^9^/L) and age (years) among stroke patients (n = 90).

	Total leucocyte count	Age
Mean	11.9512	61.11
Median	11.2000	65.00
Mode	11.00	65
Std. deviation	4.61861	15.093

**Table-II:** Results of the one-sample t-test comparing the mean total leucocyte count (TLC) in stroke patients (n = 90) with the reference mean value of 7 ×10^9^/L.

Test value=7				95%confidence interval of the difference		
	t	df	P-value	Mean difference	Lower	Upper
Total leucocyte count	10.170	89	.000	4.95122	3.9839	5.9186

The chi-square test showed no significant association between TLC category (normal vs elevated) and stroke type (ischemic vs hemorrhagic) (χ² = 0.71, df = 1, p = 0.399), indicating that elevated TLC was not associated with stroke type in this study.

One-way ANOVA demonstrated no significant difference in mean TLC among the three age groups (F = 2.186, df = 2, 87, p = 0.119), suggesting that leukocyte levels were comparable across all age groups. Pearson correlation analysis also showed no significant correlation between age and TLC (r = −0.108, p = 0.313), indicating that leukocyte count was independent of age in the study population.

## DISCUSSION

In this prospective study of 90 ED stroke patients, the mean leukocyte count was markedly elevated, reflecting the acute systemic inflammatory response to cerebrovascular injury. Acute stroke triggers robust systemic inflammation with early leukocyte mobilization and brain infiltration. For example, Simats and Liesz note that “stroke promotes systemic inflammation,” which contributes to further vascular injury^13^. Similarly, Zhu et al. emphasize that a persistent inflammatory response after ischemia exacerbates brain damage.^14^ Thus, our finding of leukocytosis compared to the normal reference value is consistent with the well-known role of inflammation in stroke pathophysiology.

We found no significant difference in TLC between ischemic and hemorrhagic stroke patients. This suggests that both stroke types can elicit a comparable leukocytic response. Some evidence supports this: many studies report robust immune activation in both ischemic and hemorrhagic strokes. However, results in the literature are mixed. For instance, Mardani et al. reported significantly higher TLC in hemorrhagic stroke (mean 11.4×10^9/L) than in ischemic stroke (9.5×10^9/L, p=0.021. The discrepancy may stem from differences in sample size, patient selection, or timing of blood sampling. In our cohort, the lack of a subtype difference may indicate that early leukocytosis is a general response to cerebral injury, regardless of hemorrhage.

Leukocyte count was also independent of age in our study. Aging is associated with immunosenescence and higher baseline inflammatory markers (e.g. CRP, IL-6), which might suggest older patients have a heightened inflammatory state. Indeed, Seah et al. note that age-related chronic inflammation can worsen stroke outcomes.^15^ However, in the setting of acute stroke, the inflammatory surge appears to be driven mainly by the insult rather than chronological age. Our finding of no correlation between age and TLC is consistent with the idea that stroke severity and acute immune activation dominate leukocyte dynamics. In other words, even if older patients have higher baseline inflammation, the immediate post-stroke leukocyte response was similar across age groups.

Importantly, leukocytosis has well-established prognostic significance in stroke. Prior studies have consistently shown that higher admission WBC counts predict more severe deficits and poorer outcomes. For example, Pinzon and Veronica found that leukocyte count was an independent predictor of greater stroke severity and unfavorable three months functional outcome.^16^ In a large randomized stroke cohort, Barow et al. observed that elevated WBC on admission was associated with lower odds of a good recovery, larger infarct volumes, and more hemorrhagic transformations.^17^ Similarly, among ischemic stroke survivors, Vo TP et al. reported that elevated WBC at admission was linked to higher 10-year rates of death and recurrent vascular events.^10^ In intracerebral hemorrhage, higher admission WBC also predicts mortality: He et al. found that patients with leukocytosis had significantly greater 90-day mortality.^18^ In reperfused stroke patients, likewise showed that leukocytosis independently predicted three month disability and death after IV thrombolysis. Thus, although in our study TLC did not differentiate stroke subtype or patient age, its elevation is nonetheless aligned with an aggressive disease course.

Overall, our findings reinforce the concept that elevated leukocyte count is a universal marker of acute stroke-related inflammation but is not specific for ischemic versus hemorrhagic etiology. Routine TLC measurement is inexpensive and readily available; while it cannot by itself distinguish stroke type, it remains a useful prognostic biomarker. Recent work suggests combining TLC with other indices (e.g. neutrophil-to-lymphocyte ratio) may further improve risk stratification. Even large population studies have linked higher WBC to stroke risk: for example, a Chinese cohort found that individuals with higher baseline WBC and neutrophil counts had significantly elevated risk of fatal stroke.^19^ Future multicenter studies with serial leukocyte measures and functional follow-up are warranted to clarify how dynamic changes in leukocytosis relate to recovery and long-term outcomes in stroke patients.

The absence of a significant association between TLC and stroke subtype in our study may be explained by the fact that both ischemic and hemorrhagic strokes initiate a similar acute systemic inflammatory response immediately after cerebral injury. In addition, the relatively small sample size may have limited the statistical power to detect subtle differences between the two stroke subtypes. Despite this, the consistently elevated TLC observed in our patients remains clinically important, as it reflects the underlying inflammatory response to acute stroke and may help identify patients at increased risk of severe disease and adverse clinical outcomes. Therefore, TLC should be considered as a useful adjunctive prognostic biomarker rather than a diagnostic marker for distinguishing stroke subtypes.

This study provides prospective local evidence that leukocytosis is a common finding in acute stroke but does not differ significantly between stroke subtypes. Clinically, it supports the use of total leukocyte count as a simple and readily available inflammatory biomarker in the emergency department.

### Strength:

A major strength of this study is its prospective design. Further large multicenter studies are needed to confirm these findings and establish the prognostic value of leukocytosis in acute stroke.

### Limitations:

A limitation of this study is that only female patients were included. Although this approach reduced potential confounding due to sex-related biological differences in inflammatory responses, the findings may not be generalizable to male stroke patients.^20^ Future studies including both sexes are needed to validate these results. In addition, no control group was included, stroke severity was not assessed, and residual confounding cannot be excluded. Therefore, larger multicenter studies with more diverse populations are needed to validate these findings. Another limitation of this study is that clinically important outcome measures, including stroke severity (NIHSS), infarct volume, functional outcome, mortality, and in-hospital complications, were not assessed.

## CONCLUSION

In this study, stroke patients demonstrated a significantly higher mean total leukocyte count compared to international reference values, indicating a strong inflammatory response associated with acute stroke. However, leukocyte levels did not differ significantly between ischemic and hemorrhagic stroke types, nor across different age groups. No meaningful correlation was found between age and TLC. These findings suggest that while elevated TLC is common in stroke, it may not be useful for distinguishing stroke subtype or predicting patient characteristics. Further larger-scale studies are recommended to explore the prognostic value of TLC in stroke management within our population.

### Recommendations:

Future studies should include these variables to better determine the prognostic significance of leukocytosis in acute stroke.
